# Dissecting Cellulitis of the Scalp: A Case Report

**DOI:** 10.7759/cureus.101164

**Published:** 2026-01-09

**Authors:** John Delgado, Micah Pippin, Robert Campbell

**Affiliations:** 1 Family Medicine, Louisiana State University Health Sciences Center, Alexandria, USA; 2 Family Medicine, Rapides Regional Medical Center, Alexandria, USA

**Keywords:** dissecting cellulitis of scalp, oral isotretinoin, scarring alopecia, skin disease/dermatology, skin of color dermatology

## Abstract

Dissecting cellulitis of the scalp, also commonly referred to as dissecting folliculitis, is a rare, chronic, relapsing, suppurative disease characterized by painful nodules, purulent drainage, burrowing, and interconnecting abscesses. Dissecting cellulitis disproportionately affects Black males in early and mid-adulthood and may result in cicatricial alopecia, scarring disfigurement, and impaired quality of life. This narrative introduces a representative case of dissecting cellulitis in an adult Black male, summarizes the epidemiology, etiology, pathology, and evaluation of the condition, reviews evidence-based best practices for management and interdisciplinary consultation, and highlights racial disparities associated with the disorder. By shedding light on dissecting cellulitis, this case report aims to facilitate early diagnosis, optimal management, and favorable outcomes for this progressive and debilitating condition.

## Introduction

Dissecting cellulitis of the scalp, also known as perifolliculitis capitis abscedens et suffodiens, was first described by Spitzer in 1903 and officially named by Hoffmann in 1908. Together with acne conglobata and hidradenitis suppurativa/acne inversa, it makes up the follicular occlusion triad/acne triad, or with pilonidal sinus, the follicular occlusion tetrad/acne tetrad [1]. The disorder is a chronic and relapsing inflammatory condition of the hair follicles, typically afflicting young men of African descent in the third to fourth decade of life [2]. Painful nodules, sterile abscesses, draining sinus tracts, and progression to cicatricial alopecia are characteristic of a typical dissecting cellulitis presentation [1,2]. This case study provides a detailed description of an African American male with a classic manifestation of dissecting cellulitis, reviews current evaluation and management principles, discusses health disparities, and advocates for early recognition and treatment to avoid irreversible disfigurement and associated psychosocial morbidity.

## Case presentation

The patient was a 63-year-old African American male who presented for evaluation of scalp lesions. Eight months previously, he developed what appeared to be a boil on the right, lateral aspect of his scalp. He noted swelling, pain, tightness, and warmth over the apparent boil or cyst. Over the following months, he noticed changes to the shape of his posterior scalp and would later develop discharge, which left his pillowcase bloodied in the mornings. As the eruption progressed and the lesions multiplied, he reported hair loss in the proximity of the outbreak. He was referred to our family medicine dermatology clinic for evaluation and treatment.

On initial presentation, the patient reported continued discomfort from the lesions with worsening drainage and alopecia. He discussed how the condition had caused him to be self-conscious and was affecting his confidence. He had never experienced these lesions prior to the initial outbreak and had not undergone any treatment. He denied any fever, chills, night sweats, weight loss, or other constitutional symptoms suggestive of infection or systemic disease. He had a past medical history of type two diabetes mellitus for which he was taking metformin 500 mg oral tablets daily. Other dermatologic conditions he had previously experienced included an axillary abscess and superficial candidiasis. He had a history of an unprovoked lower extremity deep vein thrombosis treated with apixaban (Eliquis) 5 mg daily, which he was taking indefinitely. He also reported a history of gout and generalized anxiety disorder. His surgery history included incision and drainage of an isolated abscess and an inguinal hernia repair. His family history was only notable for type two diabetes mellitus in his mother. He did not endorse alcohol, tobacco, or illicit drug use and reported no known drug allergies.

Recorded vital signs on his first visit included a temperature of 37°C (98.8°F), blood pressure of 136/98 mm Hg, heart rate of 96 beats per minute, and respiratory rate of 22 breaths per minute. The patient weighed 88.9 kg (196 lb) and had a body mass index of 27.33 kg/m^2^.

**Figure 1:**
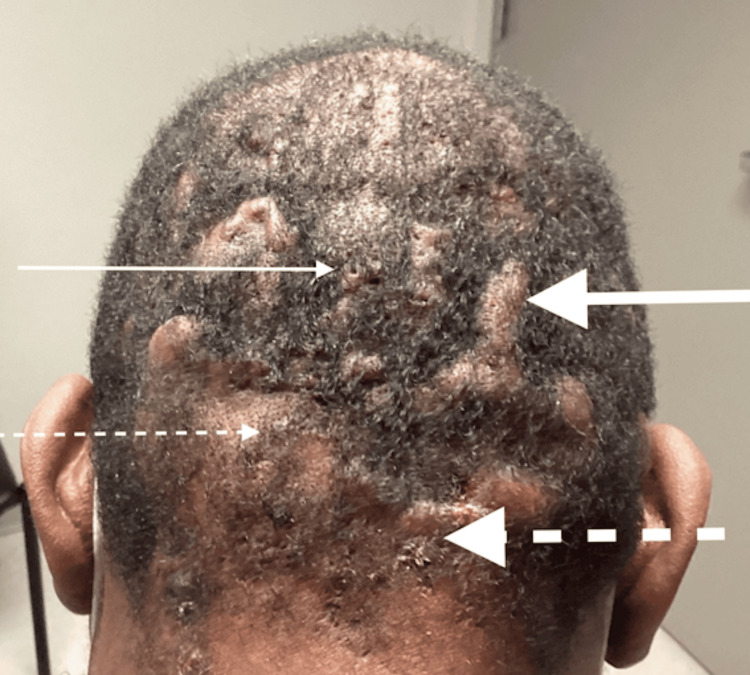
Initial presentation with sinus tract formation (thin solid arrow), keloids (thick solid arrow), alopecia (thin dashed arrow), and exudative lesions (thick dashed arrow)

On physical examination, the occiput displayed numerous tender nodulocystic lesions with sinus tract formation, malodorous discharge, keloid formation, and cicatricial scarring alopecia (Figure 1).

The affected area did not demonstrate any surrounding erythema or temperature change, and there was no associated lymphadenopathy. There were no similar outbreaks in other anatomical locations, and the axilla and groin were free from any lesions. The patient's exam was otherwise unremarkable with normal cardiac, respiratory, abdominal, and musculoskeletal findings.

**Figure 2:**
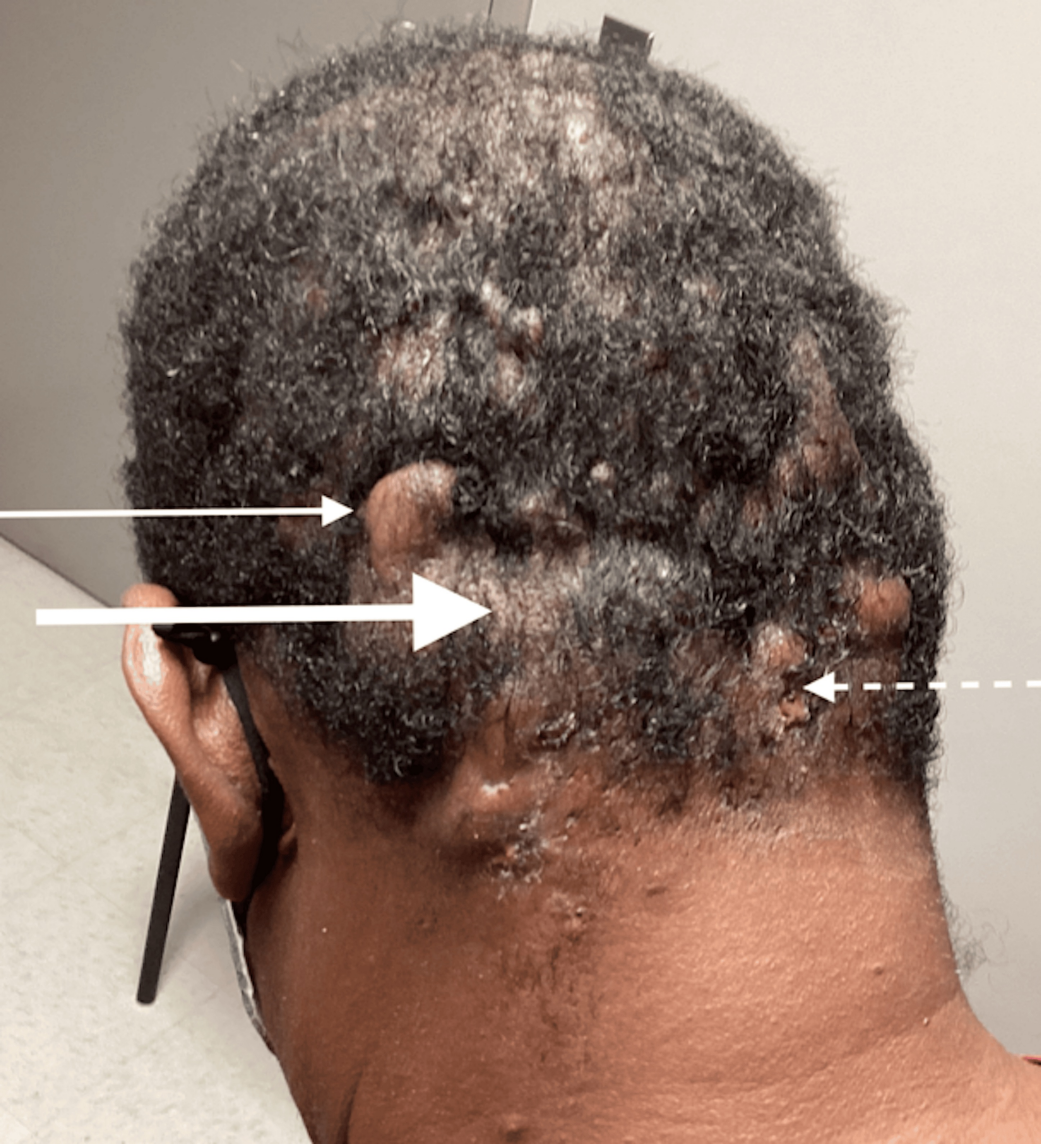
Post-doxycycline follow-up visit with continued keloid formation (thin solid arrow), alopecia (thick solid arrow), and exudative lesions (thin dashed arrow)

The patient was clinically diagnosed with dissecting cellulitis of the scalp, and treatment was initiated, including general hygiene measures, daily topical chlorhexidine cleansing, oral doxycycline 100 mg twice daily for 90 days, and zinc sulfate supplementation. The patient's two-month follow-up visit revealed only minimal improvement (Figure 2).

**Figure 3:**
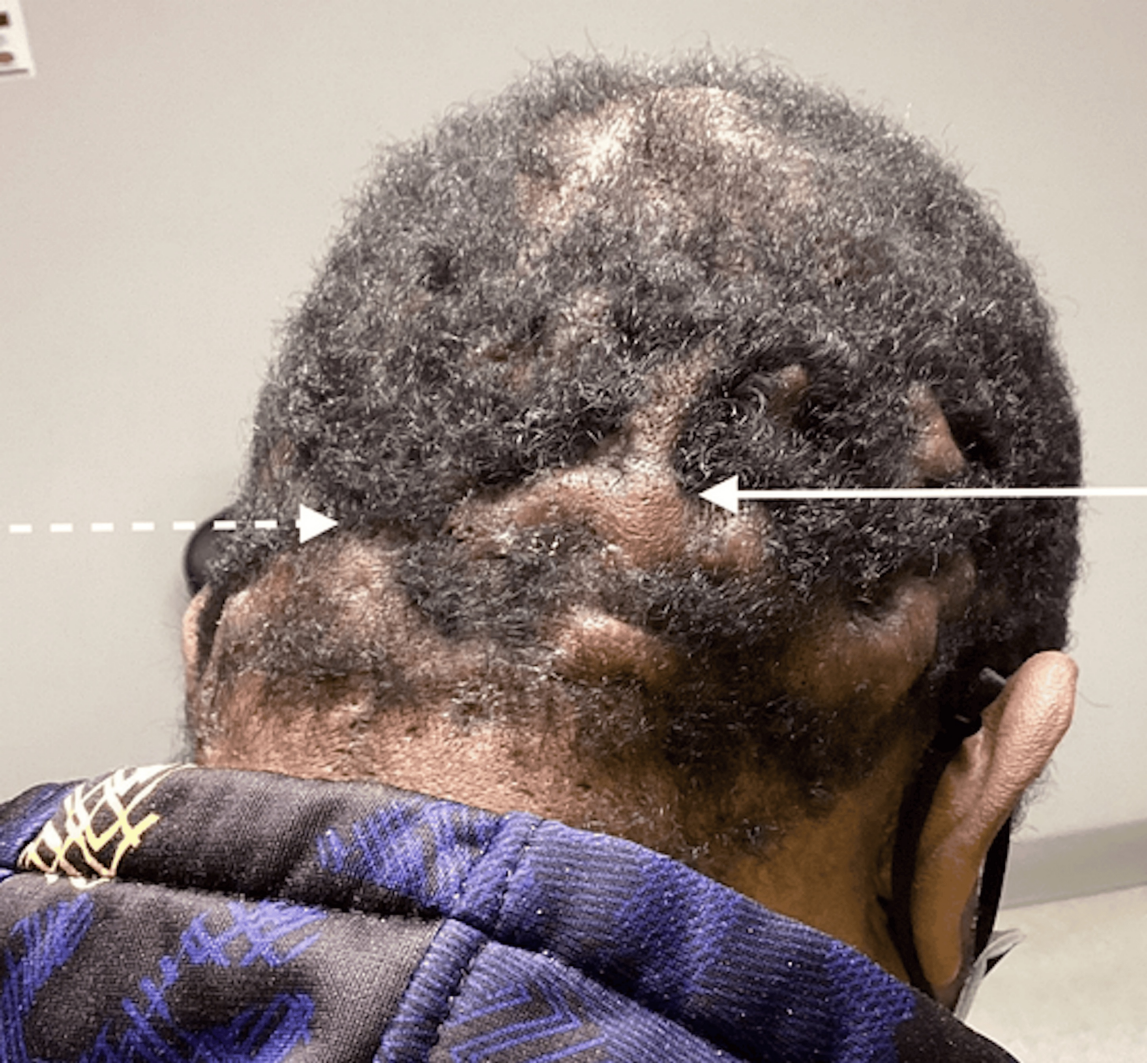
Post-isotretinoin therapy follow-up visit with continued keloid formation (solid arrow) and mildly improved alopecia (dashed arrow)

Monthly intralesional steroid injections with triamcinolone were similarly ineffective over the next four months. The patient was subsequently started on systemic isotretinoin therapy and initially reported significant improvement; however, side effects, such as dry skin, limited patient compliance. Throughout this isotretinoin regimen, the patient was seen monthly, and his lipids and liver function were monitored periodically. He did have occasional intralesional steroid injections and incision and drainage procedures when presenting with flared, painful lesions. The patient eventually received a cumulative dose of 150 mg/kg of isotretinoin over nine months, with intermittent improvement, but the treatment was ultimately ineffective overall (Figure 3).

It was decided to refer the patient to a facility with more available advanced therapies, including potential tumor necrosis factor inhibitors, laser ablation, or surgical excision.

## Discussion

Dissecting cellulitis of the scalp presents clinically as relapsing, suppurative, tender scalp nodules that eventually form draining sinuses, leading to subsequent scarring and alopecia [3]. This disease process typically involves African American males in their third to fourth decade of life, with a prevalence of approximately 0.3-4% reported in several studies [3,4]. Risk factors for the development of dissecting cellulitis of the scalp include family history, current tobacco smoking, African descent, and obesity [5]. Associations with sternoclavicular hyperostosis, polyarticular arthritis, and HLA-B27 seronegative spondyloarthropathy have been described in the medical literature [6]. An association with the keratitis-ichthyosis-deafness syndrome has also been reported, suggesting a possible interaction with connexin 26 activity [7].

Dissecting cellulitis of the scalp has the potential for a broad differential diagnosis spanning from carbuncles, furuncles, tuberculous abscess, acne keloidalis nuchae, kerion, discoid lupus erythematosus, noduloulcerative syphilis, pseudopelade of Brocq, folliculotropic mycosis fungoides with large-cell transformation, cutis verticis gyrata, lichen planopilaris, folliculitis (spinulosa) decalvans, or erosive pustular dermatosis of the scalp [8]. Dissecting cellulitis shares significant clinical overlap with hidradenitis suppurativa, as both syndromes are typically characterized by a series of chronic, inflammatory skin lesions. Dissecting cellulitis, hidradenitis, acne conglobata, and pilonidal cysts comprise the constellation of syndromes known as the follicular occlusion triad or tetrad [9]. The pathogenesis of dissecting cellulitis is believed to involve epithelial shedding as the inciting event, leading to hyperkeratosis, hair follicle dilatation, subsequent rupture, and a pathologic inflammatory response that forms nodules and abscesses, which ultimately coalesce into fistulous tracts and scarring [10].

Histological descriptions of dissecting cellulitis exhibit a spectrum of changes evident in early versus late-stage lesions, with the presence of dense neutrophilic, lymphocytic, histiocytic, and plasma cellular infiltrates in the initial stages and chronic granulomas consisting of lymphocytes, plasma cells, and foreign-body giant cells in the later stages associated most frequently with scarring and fibrosis [11]. Trichoscopy descriptions have included the presence of three-dimensional (3D) yellow dots overlying dystrophic hairs, large yellow amorphous areas, and pinpoint white dots with a whitish halo as the predominant findings [12]. Dermoscopy demonstrates characteristics of alopecia areata, including black dots, broken hairs, and yellow dots in the early phases of the disease process, evolving into cicatricial alopecia with white areas lacking follicular openings, and the characteristic feature of cutaneous clefts with hair tufts [12]. Most studies investigating a potential microbiologic etiology have shown that scalp abscesses in dissecting cellulitis are sterile. Still, needle aspiration and cultures of discharged pus have revealed organisms such as *Prevotella intermedia*, *Peptostreptococcus asaccharolyticus*, and coagulase-negative staphylococci [13,14]. The significance of these studies remains to be determined, whether indicating the presence of superinfection that exacerbates the disease process or serving as a necessary and sufficient precursor for the development of pathology [13,14].

The diagnosis of dissecting cellulitis of the scalp is predominantly clinical. Laboratory evaluation is largely non-contributory. As described, cultures are often sterile; however, exudate evaluation may assist in diagnosing secondary bacterial infection [14]. Diagnostic imaging is not routinely required for diagnosis; however, ultrasound, computed tomography (CT), or magnetic resonance imaging (MRI) may be useful for assessing deep structures, such as abscesses, or for mapping deep sinus tracts prior to surgical intervention [14].

Multiple treatment modalities exist for the management of dissecting cellulitis. Oral retinoids, such as isotretinoin, are the most extensively researched systemic agents, and effective response rates have been reported in systematic reviews and large retrospective cohorts [8,15]. The dosing regimen for isotretinoin is often cited as 0.3-1 mg/kg/day, and treatment courses for isotretinoin are typically prolonged, requiring months of therapy [15,16]. Relapses following discontinuation are often documented [15]. Oral antibiotics such as tetracyclines and macrolides are widely used, with the highest success rates observed with doxycycline [17]. These oral antibiotic courses are also often protracted, with regimens lasting up to 18 months [17]. Partial response and relapse following discontinuation are also commonly observed at follow-up. Topical regimens are generally not favored as effective disease-modifying therapeutics but may serve an adjunctive role to systemic therapy [18,19]. Topical antibiotics, antimicrobial washes, corticosteroids, and keratolytics may reduce superficial bacterial load, diminish inflammation, decrease odor and drainage, and prevent secondary infection when used as supportive adjuncts to systemic treatments [8,18]. Anti-tumor necrosis factor (TNF)-alpha biologics, such as adalimumab, may be considered for refractory cases, especially when concomitant hidradenitis suppurativa is present [20]. For acute flares, short courses of oral steroids or intralesional steroids may reduce acute inflammation and pain and serve as a bridge to more definitive therapy [8,16,18]. Incision and drainage can also be utilized to provide temporary relief for discrete abscesses but does not alter the underlying disease process or improve the syndrome's progression [8,18]. Surgical excision or resection and laser epilation are options for refractory or end-stage disease that is not responsive to primary medical therapy [8,20-24]. Lifestyle modification, such as smoking cessation and weight loss, is reasonable and often recommended; however, there is no direct evidence of effect on disease trajectory [4,8,21]. Complementary therapy with zinc supplementation has been suggested as a low-risk natural adjunctive treatment, but evidence is limited [4,8]. Many non-evidence-based supportive treatment modalities for dissecting cellulitis are extrapolated from known hidradenitis suppurativa therapeutics.

The clinical course for dissecting cellulitis is chronic and relapsing [8]. Early recognition and appropriate management can limit progression; however, sinus tract formation and scarring alopecia generally herald a permanent stage of the disease trajectory that cannot be reversed [16-18]. Complications of dissecting cellulitis include cicatricial alopecia, secondary bacterial infections, and, in rare cases, deep infections, including osteomyelitis [4,8,20]. A rare but potentially serious complication includes the development of cutaneous squamous cell carcinoma arising from chronically inflamed areas [4,8].

Primary care has a central role in managing dissecting cellulitis while also addressing related comorbidities, particularly associated metabolic syndrome elements such as obesity, diabetes, and dyslipidemia. Family physicians are ideally placed to support medication monitoring, weight-loss interventions, smoking cessation, and care coordination among the interdisciplinary team. Dermatology and surgical referrals are prudent and may provide advanced evaluation and management options for severe or refractory disease [4,8]. If comorbid inflammatory arthropathies are suspected, rheumatologic consultation is merited [8]. Dissecting cellulitis is associated with impaired quality of life and psychosocial distress, and behavioral health may be an integral component of the interdisciplinary team [4,25,26]. Rates of comorbid anxiety and depression have been reported in 21-27% of patients with dissecting cellulitis and disproportionately affect individuals with dark skin [26].

Dissecting cellulitis' disparate impact on darker-skinned patients, especially Black males, is well documented [2,26]. While the exact mechanism underlying this disproportionate effect remains unclear, several biological and health-system factors have been proposed. Tightly curled hair is associated with differences in follicular structure, which may result in an increased propensity for follicular occlusion and rupture [2,26]. Close shaving and other grooming practices may result in chronic and recurring scalp trauma, predisposing patients with darker skin to the condition [2]. Dissecting cellulitis' pathophysiologic overlap with hidradenitis suppurativa and other follicular occlusion disorders with racial prevalence and historical diagnostic delay may contribute to the disparity [2,26]. Underrecognition of inflammatory scalp disease in darker-skinned patients and delayed access to specialty care could also underscore the phenomenon of advanced disease presentations and high rates of scarring alopecia [2,8,26]. More studies are required to isolate the causative biological, behavioral, and systemic variables resulting in elevated disease burden in patients of color and optimize management strategies.

## Conclusions

Dissecting cellulitis is a destructive follicular occlusion disorder with a chronic and relapsing course predominantly affecting adult males of African ancestry. Long-term sequelae of the condition include scarring alopecia and disfiguring inflammatory remodeling of the posterior scalp. The diagnosis is primarily clinical and is supported by heightened suspicion in patients at risk. Early recognition and appropriate disease-modifying interventions are paramount to avoid not only dermatologic complications but also psychosocial pathology commonly associated with the condition. Systematic reviews and large retrospective cohorts have identified oral isotretinoin as a key pharmacologic therapy for dissecting cellulitis, alongside systemic antibiotics, corticosteroids, biologics, lasers, and surgical interventions, each fulfilling specific roles based on disease severity and chronicity. An emphasis on prompt diagnosis, optimized therapies, interdisciplinary and specialist access, and culturally competent counseling is necessary to facilitate improved outcomes in patients with dissecting cellulitis, especially disproportionately affected individuals with skin of color.

## References

[1] Lee CN, Chen W, Hsu CK, Weng TT, Lee JY, Yang CC (2018). Dissecting folliculitis (dissecting cellulitis) of the scalp: a 66-patient case series and proposal of classification. J Dtsch Dermatol Ges.

[2] Scott DA (1988). Disorders of the hair and scalp in blacks. Dermatol Clin.

[3] Jerome MA, Laub DR (2014). Dissecting cellulitis of the scalp: case discussion, unique considerations, and treatment options. Eplasty.

[4] Jaroonwanichkul S, Rajpara A (2024). Dissecting cellulitis of the scalp. Kans J Med.

[5] Cuellar TA, Roh DS, Sampson CE (2020). Dissecting cellulitis of the scalp: a review and case studies of surgical reconstruction. Plast Reconstr Surg Glob Open.

[6] Tran AX, Lefante JJ, Murina A (2022). Risk factors for dissecting cellulitis of the scalp: a case-control study. J Am Acad Dermatol.

[7] Bjellerup M, Wallengren J (1990). Familial perifolliculitis capitis abscedens et suffodiens in two brothers successfully treated with isotretinoin. J Am Acad Dermatol.

[8] Prasad SC, Bygum A (2013). Successful treatment with alitretinoin of dissecting cellulitis of the scalp in keratitis-ichthyosis-deafness syndrome. Acta Derm Venereol.

[9] Scheinfeld N (2014). Dissecting cellulitis (perifolliculitis capitis abscedens et suffodiens): a comprehensive review focusing on new treatments and findings of the last decade with commentary comparing the therapies and causes of dissecting cellulitis to hidradenitis suppurativa. Dermatol Online J.

[10] Vasanth V, Chandrashekar BS (2014). Follicular occlusion tetrad. Indian Dermatol Online J.

[11] Chung-Min Y, Jung-Ha K, Song-Hee H, Ji-An C (2021). Follicular occlusion triad: a case report. J Wound Manag Res.

[12] Sperling LC (2001). Scarring alopecia and the dermatopathologist. J Cutan Pathol.

[13] Rakowska A, Slowinska M, Kowalska-Oledzka E (2012). Trichoscopy of cicatricial alopecia. J Drugs Dermatol.

[14] El Sayed F, Ammoury A, Dhaybi R, Aftimos G, Bazex J (2006). Perifolliculitis capitis abscedens et suffodiens: an unusual case triggered by trauma. J Eur Acad Dermatol Venereol.

[15] Ramesh V (1990). Dissecting cellulitis of the scalp in 2 girls. Dermatologica.

[16] Melo DF, Trüeb RM, Dutra H, Lima MM, Machado CJ, Dias MF (2020). Low-dose isotretinoin as a therapeutic option for dissecting cellulitis. Dermatol Ther.

[17] Guo W, Zhu C, Stevens G, Silverstein D (2021). Analyzing the efficacy of isotretinoin in treating dissecting cellulitis: a literature review and meta-analysis. Drugs R D.

[18] Karpouzis A, Giatromanolaki A, Sivridis E, Kouskoukis C (2003). Perifolliculitis capitis abscedens et suffodiens successfully controlled with topical isotretinoin. Eur J Dermatol.

[19] Abdennader S, Vignon-Pennamen MD, Hatchuel J, Reygagne P (2011). Alopecic and aseptic nodules of the scalp (pseudocyst of the scalp): a prospective clinicopathological study of 15 cases. Dermatology.

[20] Masson R, Jeong CY, Ma E (2023). Treatments for dissecting cellulitis of the scalp: a systematic review and treatment algorithm. Dermatol Ther (Heidelb).

[21] de Melo WC, Avci P, de Oliveira MN (2013). Photodynamic inactivation of biofilm: taking a lightly colored approach to stubborn infection. Expert Rev Anti Infect Ther.

[22] Krasner BD, Hamzavi FH, Murakawa GJ, Hamzavi IH (2006). Dissecting cellulitis treated with the long-pulsed Nd:YAG laser. Dermatol Surg.

[23] Bechara FG, Podda M, Prens EP (2021). Efficacy and safety of adalimumab in conjunction with surgery in moderate to severe hidradenitis suppurativa: the SHARPS randomized clinical trial. JAMA Surg.

[24] Baneu NS, Bloancă VA, Szilagyi D (2021). Surgical management of dissecting cellulitis of the scalp using free latissimus dorsi flap and meshed split-thickness skin graft: a case report. Medicine (Baltimore).

[25] Gerlero P, Peron I, Doche I, Freitas Rodrigues E, Macedo T, Rivitti-Machado MC (2025). Dissecting cellulitis of the scalp: clinical characteristics and impact on quality of life of 66 Brazilian patients. An Bras Dermatol.

[26] Ghanshani R, Chung CS, Park SE (2025). Dissecting cellulitis of the scalp: a retrospective cohort study of demographics, provider landscape, and comorbidities. Int J Dermatol.

